# Self-harm and suicide risk amongst attendees at five lower courts in London, England

**DOI:** 10.1192/j.eurpsy.2025.398

**Published:** 2025-08-26

**Authors:** J. McCarthy, E. Chaplin, C. Allely, A. Forrester

**Affiliations:** 1 London South Bank University, London; 2 University of Salford, Manchester; 3 Cardiff University, Cardiff, United Kingdom

## Abstract

**Introduction:**

Individuals in contact with the criminal justice system are at higher risk of suicide than the general population (Carter et al. EClinicalMedicine 2022, 44, 101266). Research to date has concentrated on the prison population with little evidence on the risk of suicide and self-harm for those defendants within the Court system including those referred to the Court Mental Health Liaison and Diversion Services. Court Mental Health Liaison and Diversion services were developed in England to support vulnerable people when they first come into contact with the criminal justice system.

**Objectives:**

The main aim of the study was to analyse the existing service data to examine rates of self-harm behavior and suicide ideation of those defendants presenting to the Court Mental Health Liaison and Diversion Services across five Magistrates Courts (lower courts) in London, England. In addition, a further aim was to establish if risk factors such as mental illness and substance misuse but also other vulnerabilities such as neurodevelopmental disorders are associated with the risk for self-harm behaviour or suicide along with demographic factors of age, gender and ethnicity.

**Methods:**

The study analysed service level data of five London Magistrates’ Courts covering a timeframe from September 2015 to April 2017. During this time 9088 attendees were referred to the Court Mental Health & Liaison Diversion service covering the five courts. Attendees were screened for current risk of suicide ideation and self-harm behaviour as part of the mental health assessment. Data examined was from the National Health Service (NHS) minimum mental health data set which reflects current clinical and custody records and is obtained from frontline court and health service staff.

**Results:**

An overall rate of 14.2% for self-harm behaviour and/or suicide ideation was found for attendees presenting to five London Court Liaison and Diversion Services over a 20-month time frame. Aside from autism and bipolar affective disorder, the current large study showed a significant association between self-harm behaviour and suicide ideation with several mental disorders. The study found no significant differences for risk of self-harm behaviour and suicide ideation relating to gender or ethnicity.

**Conclusions:**

This group of defendants presented with high levels of severe mental illness, substance and alcohol misuse and neurodevelopmental disorder which increased the individual vulnerability to express suicidal ideation as has been found in smaller studies. The wider criminal justice services need to examine the current approach to screening for risk of suicide ideation and self-harm behaviour given the high rates of completed suicide within the prison population compared to the general population.

**Disclosure of Interest:**

J. McCarthy Grant / Research support from: £674,000, E. Chaplin Grant / Research support from: £674,000, C. Allely: None Declared, A. Forrester Grant / Research support from: £674,000.

